# Favorable Changes in Fasting Glucose in a 6-month Self-Monitored Lifestyle Modification Programme Inversely Affects Spexin Levels in Females with Prediabetes

**DOI:** 10.1038/s41598-019-46006-0

**Published:** 2019-07-01

**Authors:** Nasser M. Al-Daghri, Kaiser Wani, Sobhy M. Yakout, Hazim Al-Hazmi, Osama E. Amer, Syed Danish Hussain, Shaun Sabico, Mohammed Ghouse Ahmed Ansari, Sara Al-Musharaf, Amal M. Alenad, Majed S. Alokail, Mario Clerici

**Affiliations:** 1 Chair for Biomarkers of Chronic Diseases, Department of Biochemistry, College of Science, King Saud University, Riyadh, 11451 Saudi Arabia; 2 College of Applied Medical Sciences, King Saud University, Riyadh, Saudi Arabia; 3 Department of Physiopathology and Transplantation, University of Milan, via F.lli Cervi 93, Segrate, 20090 Milan Italy; 4 IRCCS Fondazione Don Carlo Gnocchi ONLUS, Milan, 20148 Italy

**Keywords:** Predictive markers, Pre-diabetes

## Abstract

Spexin (SPX) is a novel peptide thought to have a role in various metabolic regulations. Given its presumed body-weight regulatory functions, we aimed to determine whether lifestyle intervention programs on weight loss and fasting glucose (FG) improvement among people with impaired glucose regulation also alter levels of circulating SPX. A total of 160 Saudi adult males and females with prediabetes were randomly selected from a larger cohort (N = 294) who underwent a 6-month lifestyle modification program to improve their glycemic status. Participants were split into two groups based on differences in glucose levels post-intervention, with the first 50% (improved group) having the most significant reduction in FG. SPX was measured at baseline and after 6 months. Changes in SPX was significant only in the improved group [baseline: median (Q1–Q3) of 164 pg/ml (136–227) vs follow-up: 176 pg/ml (146–285); p < 0.01]. When stratified by sex, the significant increase was observed only in females [159 pg/ml (127–252) vs 182.5 (152,369.1); p < 0.01]. Furthermore, SPX levels showed a significant inverse association with FG (β = −0.22, p = 0.003) even after adjustment with age and BMI, again only in females. Circulating SPX levels increase over time in people with prediabetes, particularly women who responded favorably in a 6-month lifestyle intervention program. Whether an unknown mechanism regulating the sexual disparity seen in SPX levels post-intervention exists should be further investigated using a larger sample size.

## Introduction

Spexin (SPX) is a newly identified neuropeptide with emerging roles in many metabolic processes such as satiety, pubertal growth and reproduction^1,2^. Discovered through bioinformatics approach using Markov model screening^3^, SPX consists of 14 amino acids and belongs to spexin/galanin/kisspeptin gene family^4^. SPX is widely expressed in a number of rat tissues such as liver, testis, ovary, adrenal gland, etc., making it a promising biomarker in pathological disorders such as the metabolic syndrome, diabetes and others^5^.

SPX studies in animal models like fish and rodents revealed its role in various metabolic regulations. In 2014, Walewski et. al. showed that SPX regulates satiety in mice, possibly by inhibiting the uptake of long chain fatty acids into hepatocytes and adipocytes, resulting in weight loss^6^. Similar results confirmed the role of SPX as a regulator of fatty acid uptake in adipocytes and hepatocytes in fish^7^ and mice models^8^, respectively. Their results suggest that this neuropeptide hormone is an adipocyte-secreted body-weight control factor. In humans, recent studies revealed that the levels of circulating SPX are significantly lower in obese women and children than the lean ones, suggesting a link between SPX and body weight regulation^9,10^. Despite the emerging evidence on the role of SPX in many physiological processes, knowledge on the functions of SPX, its expression, secretion, and influence on tissues, at least in humans, are still in its preliminary stages. However, taking into account SPX’s metabolic role, it is interesting to investigate its functions in insulin resistance (IR) and glucose homeostasis in type 2 diabetes mellitus (T2DM).

T2DM is associated with disturbances in glucose homeostasis and has a huge impact on the overall health care cost of the country. Landmark lifestyle intervention studies focusing on people with impaired glucose regulation (prediabetes) such as the Diabetes Prevention Program (DPP)^11^ and others^12,13^, revealed that progression into T2DM can be delayed. Such lifestyle intervention programs in high risk populations, done in the last two decades all over the world, focus mainly on weight loss, increased physical activity and reduction of fasting glucose. Since SPX has a possible relation with glucose homeostasis, it is interesting to study whether these lifestyle intervention programs, focusing on weight loss; increased physical activity and reduction of fasting glucose also modify levels of SPX. The present study aimed to observe changes in SPX levels over time after 6 months of lifestyle modification program given to adult men and women with pre-diabetes. Additionally, we investigated the relationship of SPX with fasting glucose and other glycemic indices in these participants.

## Results

### Characteristics of Groups across Time Points

Table 1 shows the anthropometrics and biochemical characteristics of the study participants at baseline and end of the study. 160 subjects were divided into two groups: non-improved group (N = 80%, change in FG = median (IQR) = 1.57 (3.4); mean age = 43.37 ± 8.8 years) and improved group (N = 80%, change in FG = −22 (10.6); mean age = 43.32 ± 9.4 years). The table also shows the differences between the two groups at baseline. Baseline anthropometric and other measured biochemical characteristics were not statistically significant in both groups (depicted by P^A^ in Table 1) except in fasting glucose (6.31 ± 0.5 mmol/l in non-improved group vs 6.14 ± 0.4 mmol/l in the improved group, p = 0.02).

**Table 1:** Baseline and Follow-Up Characteristics of Participants According to Groups.

Parameters	Non-improved Group		Improved Group		P^A^
	Baseline	Follow-Up	Baseline	Follow-Up	
% Change in FG	1.57 (3.4)		−22.00 (10.6)		—
Age (years)	43.37 ± 8.8		43.32 ± 9.4		0.97
Weight (kg)	81.1 ± 13.0	82.51 ± 13.2	79.83 ± 14.8	78.4 ± 14.3**	0.55
BMI (kg/m^2^)	31.5 ± 5.2	32.1 ± 5.8	31.3 ± 5.5	30.7 ± 5.5*	0.79
Waist (cm)	102.2 ± 12.3	103.0 ± 13.2	99.2 ± 13.3	98.4 ± 13.7*	0.19
Hips (cm)	110.6 ± 11.4	110.3 ± 10.1	107.5 ± 10.8	107.1 ± 10.8	0.20
SBP (mmHg)	124.2 ± 12.5	125.8 ± 16.4	123.2 ± 16.0	120.3 ± 19.7	0.70
DBP (mmHg)	77.0 ± 9.0	78.3 ± 10.8	78.6 ± 11.0	77.0 ± 11.9	0.27
FG (mmol/l)	6.3 ± 0.5	6.3 ± 0.5	6.1 ± 0.4	5.0 ± 0.6**	**0**.**02**
Insulin (μU/ml)	16.1 (6.0,27.9)	13.9 (6.8,28.5)	18.0 (5.7,30.3)	14.4 (6.1,27.5)	0.70
HbA1c (%)	5.9 ± 0.8	6.2 ± 1.1	5.77 ± 0.5	4.98 ± 1.5**	0.35
HOMA-IR	4.2 (1.7,8.3)	3.8 (1.9,8.1)	4.95 (1.7,8.6)	3.13 (1.4,6.2)**	0.85
QUICKI	0.55 ± 0.1	0.56 ± 0.1	0.58 ± 0.2	0.65 ± 0.2*	0.49
McAuley ISI	5.72 (4.4,7.1)	6.12 (4.4,6.9)	5.40 (4.3,7.4)	5.96 (4.5,7.7)	0.65
TG (mmol/l)	1.6 (1.2,2.1)	1.6 (1.3,2.3)	1.63 (1.1,2.3)	1.51 (1.2,2.1)	0.80
Cholesterol (mmol/l)	4.9 ± 1.1	4.9 ± 1.1	4.82 ± 1.4	4.84 ± 1.2	0.83
HDL-C (mmol/l)	1.2 ± 0.4	1.0 ± 0.4*	1.06 ± 0.4	0.94 ± 0.3*	0.14
LDL-C (mmol/l)	2.7 (2.3,3.6)	2.9 (2.2,3.8)	2.8 (2.2,3.6)	3.13 (2.4,3.7)	0.78
SPX (pg/ml)	172 (138,198)	153 (131,190)	164 (136,227)	176 (146,285)**	0.97

Note: % change in FG (follow-up – baseline) is represented as median (Inter-quartile range). Rest of the data is presented as Mean ± SD for continuous normal variables and medians (25^th^–75^th^ percentile) for continuous non-normal variables. HOMA-IR, QUICKI and McAuley ISI are indices for insulin resitance and insulin sensitivity. Paired T-test and Wilcoxon signed-rank test was used to see differences across time points within groups; *denotes significance at p < 0.05; **denotes significant at p < 0.01 level, P^A^ represents difference two groups at baseline (calculated by independent sample t-test and Mann-Whitney U-test for Gaussian and non-Gaussian variables, respectively).

As expected, mean fasting glucose (mmol/l) in the non-improved group did not change significantly from baseline to end of study (p = 0.62). The mean fasting glucose significantly in the improved group over time (mean change of −1.19 mmol/l, p < 0.01). Correspondingly, there was a significant decrease in weight (mean change of −1.44 kgs, p < 0.01), BMI (mean change of −0.55 kg/m2, p = 0.01) and waist circumference (mean change of −0.78 cm, p = 0.04) only in the improved group. These parameters remain unchanged in the non-improved group. As expected, the glycemic indices improved significantly only in the improved group [HbA1c, HOMA-IR (p < 0.01 for both) and QUICKI (p < 0.05)]. No significant changes in glycemic indices over time were seen in the non-improved group except for HbA1c which increased significantly (p = 0.01). No significant changes were seen in the lipid indices over time in both groups except HDL-cholesterol, which decreased significantly in both groups. SPX levels [median (Q1, Q3)] increased significantly over time in the improved group [176 pg/ml (146,285) vs 164 pg/ml (136,227), p < 0.01]. No significant changes in SPX were observed in the non-improved group. Supplementary Fig. 1 shows the increase in SPX levels according to quartiles FG.

### Clinical characteristics of the groups according to sex

Table 2 shows the sex-specific changes in the clinical characteristics of both groups over time. As expected, mean fasting glucose levels decreased significantly in the improved group (mean change of −1.1 mmol/l, p < 0.01 and −1.39 mmol/l, p < 0.01 in females and males, respectively). HOMA-IR and QUICKI also improved in this group but in a sex-dimorphic manner, where HOMA-IR decreased and QUICKI increased significantly only in females. HbA1c levels in this group decreased significantly in both females (p < 0.01) as well in males (p < 0.05). All glycemic indices remain unchanged statistically in both sexes in the non-improved group.

**Table 2:** Sex-Specific Changes in Clinical Characteristics of Groups Over Time.

Parameters	Non-Improved Group [N = 80]				Improved Group [N = 80]			
	Females [N = 41]		Males [N = 39]		Females [N = 55]		Males [N = 25]	
	Baseline	6 Months	Baseline	6 Months	Baseline	6 Months	Baseline	6 Months
Weight (kg)	79.8 ± 11.6	81.5 ± 11.9	82.2 ± 14.1	83.4 ± 14.3	76.6 ± 15.2	75.4 ± 14.6	87.0 ± 11.0	85.1 ± 11.0^**^
BMI (kg/m^2^)	33.4 ± 5.2	34.1 ± 5.5	29.8 ± 4.7	30.3 ± 5.4	31.6 ± 5.8	31.1 ± 5.7	30.5 ± 4.6	29.9 ± 4.8^**^
Waist (cm)	98.4 ± 12.4	98.5 ± 13.5	106.8 ± 10.7	108.6 ± 10.8	95.3 ± 13.2	94.2 ± 13.4^*^	108.2 ± 8.7	108.3 ± 8.6
Hips (cm)	113.3 ± 8.7	112.4 ± 9.3	107.3 ± 13.4	107.9 ± 10.7	108.2 ± 11.8	108.0 ± 11.2	105.6 ± 7.4	104.4 ± 9.7
SBP (mmHg)	122.8 ± 13.7	123.7 ± 16.7	125.6 ± 11.0	128.1 ± 16.0	121.2 ± 15.1	118.4 ± 22	128.1 ± 17.2	124.8 ± 12.0
DBP (mmHg)	77.4 ± 10.6	76.3 ± 12	76.5 ± 7.0	80.3 ± 9.2	77.5 ± 11.7	76.9 ± 13.4	81.1 ± 9	77.2 ± 7.7
FG (mmol/l)	6.4 ± 0.5	6.4 ± 0.5	6.3 ± 0.5	6.3 ± 0.5	6.0 ± 0.4	4.9 ± 0.7^**^	6.4 ± 0.3	5.1 ± 0.2^**^
Insulin (μU/ml)	12.8 (6,26)	11.0 (6,17)	17.1 (12,33)	17.1 (12,31)	14.4 (5,26)	9.5 (3,17)	27.2 (15,39)	27.1 (20,43)
HbA1c (%)	5.88 ± 0.4	6.12 ± 1.1	5.87 ± 1.1	6.33 ± 1.1	5.6 ± 0.3	4.8 ± 1.2^**^	6.2 ± 0.6	5.4 ± 2.0^*^
HOMA-IR	3.5 (1.6,7.6)	3.2 (1.7,4.9)	4.6 (3,8.6)	4.8 (3.1,8.5)	3.7 (1.3,6.7)	2.2 (0.7,3.4)^**^	7.6 (3.8,11.5)	6.2 (4.5,9.3)
QUICKI	0.59 ± 0.2	0.58 ± 0.2	0.52 ± 0.1	0.53 ± 0.2	0.64 ± 0.2	0.75 ± 0.3*	0.48 ± 0.1	0.48 ± 0.1
McAuley ISI	6.46 (4.8,7.8)	6.33 (5.3,7.2)	4.80 (4.1,7.0)	4.69 (4.2,6.3)	6.04 (4.7,8.7)	6.75 (5.5,9.5)	4.45 (4.0,6.2)	4.61 (4.0,4.9)
TG (mmol/l)	1.5 (1.2,2)	1.4 (1.2,2)	1.7 (1.3,2.6)	1.9 (1.4,2.3)	1.4 (1.0,1.2)	1.4 (1.1,1.9)	2.2 (1.6,2.5)	2.0 (1.2,2.7)
Chol. (mmol/l)	5.2 ± 1.2	5.1 ± 1.2	4.6 ± 1	4.7 ± 1.0	4.8 ± 1.2	4.9 ± 1.2	4.9 ± 1.7	4.8 ± 1.3
HDLC (mmol/l)	1.2 ± 0.4	1.12 ± 0.4*	1.1 ± 0.4	1.0 ± 0.4	1.1 ± 0.4	1.0 ± 0.4^*^	0.9 ± 0.4	0.8 ± 0.2
LDLC (mmol/l)	2.9 (2.4,3.8)	3.09 (2.6,4.1)	2.6 (1.9,3)	2.65 (2.1,3.6)	2.79 (2.1,3.6)	3.12 (2.5,3.7)	2.99 (2.5,3.5)	3.25 (2.3,3.9)
SPX (Pg/ml)	172 (140,213)	157 (137,185)	168 (134,191)	142 (124,190)	159 (127,252)	182(152,369) ^**^	165 (144,205)	171 (145,199)

Note: Data presented as Mean ± SD for continuous normal variables and medians (25^th^ percentile, 75^th^ percentile) for continuous non-normal variables. HOMA-IR, QUICKI and McAuley ISI are indices for insulin resitance and insulin sensitivity. Paired samples t-test and Wilcoxon signed-rank test is used to test the differences in central tendency for continous normal and non-normal variables respectively. FG and SPX referes to fasting blood glucose and Spexin respectively. P < 0.05 is taken as significant. *Depicts p-value < 0.05 and **depicts p-value < 0.01.

Both weight and BMI improved significantly only in males in the improved group (mean changes of −1.93 kg and −0.65 kg/m^2^, both p < 0.01, respectively) while waist circumference improved only in females in this group (mean change of −1.13 cm, p < 0.01). These significant changes were not seen in the non-improved group. HDL-cholesterol decreased significantly post-intervention in both female groups over time.

Levels of circulating SPX increased significantly only in the females of the improved group over time (p < 0.01) while in males, the increase in SPX levels was not significant. The SPX levels in both males and females in the non-improved group did not significantly change over time.

**Figure 1:**
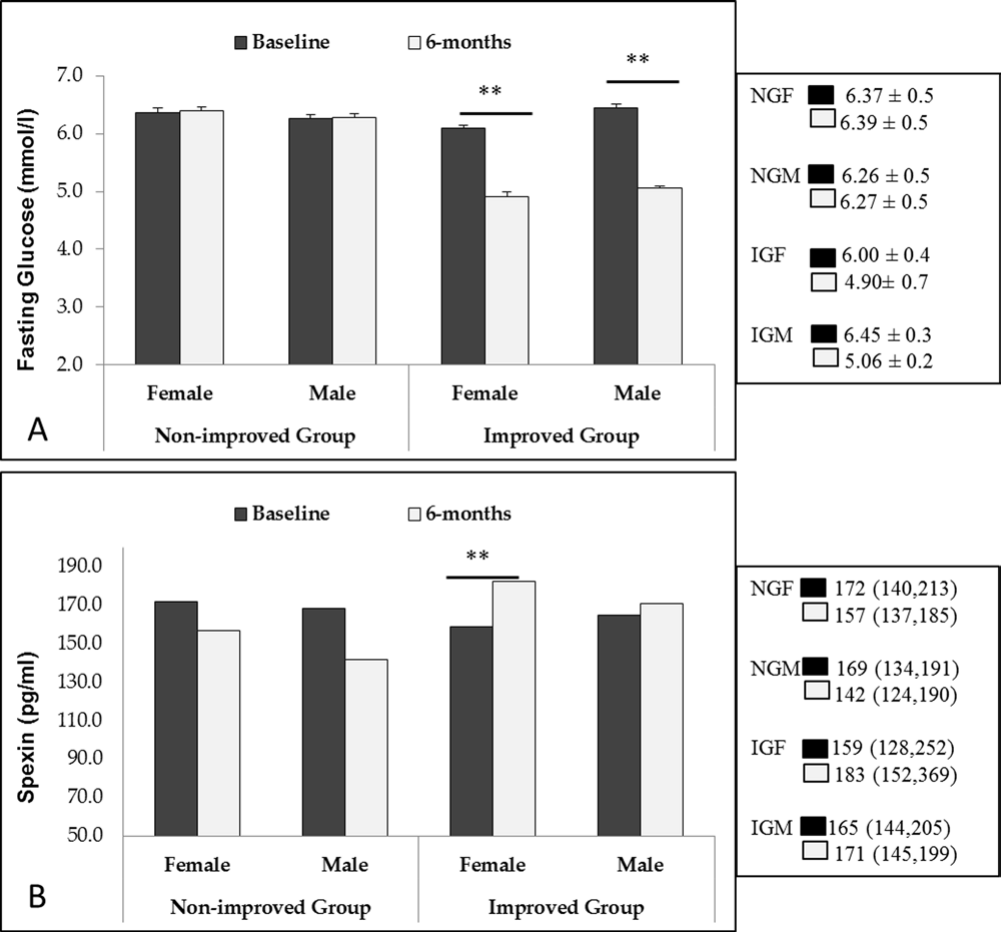
Changes in levels of fasting glucose and spexin overtime. Data presented as Mean ± SD for fasting glucose (**A**) and median (25th percentile, 75th percentile) for SPX (**B**). NG and IG represent non-improved and improved groups respectively while F and M represents females and males respectively. **Depicts p-value < 0.01.

Figure 1 shows the circulating levels of fasting glucose (1 A) and SPX (1B) in the two groups at baseline and post-intervention, according to sex. Fasting glucose reduced significantly in both males and females over time in the improved group while SPX levels increased significantly in only females.

### Intervention Effects in SPX and glycemic indices

Table 3 shows the intervention effects in the two groups stratified according to sex. As expected, fasting glucose improved significantly in both sexes in the improved group. Insulin resistance index (HOMA-IR) decreased significantly in only one of the four sub-groups (females in the improved group, mean change of −0.22, p < 0.01) and this significant decrease was also seen in females when all the subjects were taken into consideration (mean change of −0.12, p = 0.02). The insulin sensitivity index (QUICKI) also improved in females of the improved group, not in males. Lastly, SPX increased significantly over time only in females of the improved group.

**Table 3:** Intervention Effects in SPX and Glycemic Indices.

Study Groups					Intervention Effects: Mean Change, p			
	Gr 1 (N = 80)		Gr 2 (N = 80)		F (6M vs B)	M (6M vs B)	Gr 1 (6M vs B)	Gr 2 (6M vs B)
	F (N = 41)	M (N = 39)	F (N = 55)	M (N = 25)				
**Fasting Glucose**								
Baseline	6.37 (0.07)	6.26 (0.06)	6.00 (0.06)	6.44 (0.09)	**−0**.**54**, **<0**.**01**	**−0**.**69**, **<0**.**01**	0.02, 0.69	**−1**.**25**, **<0**.**01**
6-months	6.39 (0.08)	6.27 (0.07)	4.90 (0.07)	5.06 (0.10)				
6M vs B	0.03, 0.74	0.02, 0.83	**−1**.**1**, **<0**.**01**	**−1**.**4**, **<0**.**01**				
**Log insulin**								
Baseline	1.06 (0.08)	1.23 (0.08)	1.01 (0.07)	1.34 (0.09)	−0.08, 0.13	0.06, 0.34	−0.02, 0.78	−0.01, 0.94
6-months	1.03 (0.08)	1.24 (0.08)	0.89 (0.07)	1.46 (0.09)				
6M vs B	−0.03, 0.66	0.01, 0.97	−1.12, 0.08	0.11, 0.22				
**Log HOMA-IR**								
Baseline	0.51 (0.08)	0.67 (0.08)	0.43 (0.07)	0.80 (0.09)	**−0**.**12**, **0**.**02**	0.00, 0.95	−0.02, 0.79	−0.11, 0.06
6-months	0.48 (0.07)	0.67 (0.08)	0.21 (0.07)	0.81 (0.09)				
6M vs B	−0.03, 0.68	0.00, 0.98	**−0**.**22**, **<0**.**01**	0.01, 0.96				
**QUICKI**								
Baseline	0.59 (0.03)	0.52 (0.04)	0.64 (0.03)	0.48 (0.04)	**0**.**06**, **0**.**04**	0.01, 0.85	0.01, 0.76	0.05, 0.07
6-months	0.58 (0.04)	0.53 (0.04)	0.75 (0.04)	0.47 (0.05)				
6M vs B	−0.01, 0.97	0.01, 0.65	**0**.**12**, **<0**.**01**	−0.01, 0.88				
**Log SPX**								
Baseline	2.25 (0.03)	2.25 (0.03)	2.28 (0.03)	2.24 (0.04)	**0**.**06**, **0**.**04**	−0.002, 0.97	0.004, 0.90	0.05, 0.18
6-months	2.28 (0.05)	2.24 (0.05)	2.37 (0.04)	2.26 (0.06)				
6M vs B	0.03, 0.62	−0.02, 0.73	**0**.**09**, **0**.**04**	0.01, 0.84				

Note: Data presented as Mean (Standard error) for baseline and 6-months. 6 M, B, F, M, Gr1,Gr2, HOMA-IR and QUICKI represents 6-months, baseline, female, male, non-improved group, improved group, insulin resitance and insulin sensitivity index respectively. Changes at time-intervals are presented as mean change and associated p-value. Two-way repeated measures ANOVA was used for testing intervention effects. P < 0.05 is taken as significant.

### Sex-specific associations of SPX with glycemic indices at both time points

Table 4 shows the results of regression analysis using SPX as dependent variable and glycemic indices as independent variables. Post-intervention data points were used to construct this analysis. Model D was constructed after adjustments with BMI, age and fasting glucose. In all participants, circulating SPX levels showed a significant inverse association with fasting glucose levels even after adjustments for age and BMI (standardized β = −0.17, p = 0.001). Stratified according to sex, this significant association was observed only in females (β = −0.22, p = 0.003) and not in males (β = −0.09, p = 0.28). The same trend was observed with insulin and HOMA-IR, where the inverse association was significant even after adjustments for age, BMI and fasting glucose (Model D). The inverse association of SPX with HbA1c lost statistical significance after adjustment with fasting glucose values. QUICKI showed a significant positive association with SPX even after adjustments in females only (β = 0.35, p < 0.01).

**Table 4:** Regression Analysis using log SPX as Dependent Variable and Glycemic Indices as Independent Variables.

Model	All Subjects [N = 160]			Females [N = 96]			Males [N = 64]		
	β	95% CI	p	β	95% CI	p	β	95% CI	p
**GLUCOSE**									
A	−0.18	−0.09, −0.02	0.001	−0.20	−0.11, −0.02	<0.001	−0.09	−0.09,0.03	0.30
B	−0.18	−0.10, −0.03	0.001	−0.22	−0.12, −0.03	0.002	−0.09	−0.09,0.03	0.30
C	−0.17	−0.10, −0.02	0.001	−0.22	−0.12, −0.03	0.003	−0.09	−0.09,0.03	0.28
**INSULIN**									
A	−0.16	−0.15, −0.02	0.01	−0.22	−0.21, −0.03	0.008	−0.06	−0.16,0.08	0.53
B	−0.16	−0.16, −0.02	0.01	−0.25	−0.23, −0.05	0.003	−0.05	−0.15,0.09	0.59
C	−0.15	−0.15, −0.01	0.02	−0.25	−0.23, −0.05	0.003	−0.04	−0.14,0.09	0.72
D	−0.15	−0.15, −0.01	0.02	−0.28	−0.23, −0.05	0.003	−0.03	−0.14,0.10	0.78
**HOMA-IR**									
A	−0.16	−0.15, −0.02	0.01	−0.23	−0.21, −0.04	0.005	−0.06	−0.16,0.08	0.54
B	−0.17	−0.16, −0.02	0.009	−0.27	−0.23, −0.06	0.002	−0.05	−0.16,0.09	0.61
C	−0.16	−0.15, −0.02	0.01	−0.27	−0.24, −0.06	0.002	−0.03	−0.14,0.10	0.76
D	−0.15	−0.15, −0.01	0.02	−0.25	−0.23, −0.05	0.003	−0.03	−0.14,0.10	0.78
**QUICKI**									
A	0.24	0.08,0.47	<0.01	0.32	0.09,0.57	<0.01	0.02	−0.4,0.50	0.90
B	0.25	0.08,0.48	<0.01	0.35	0.13,0.60	<0.01	0.01	−0.5,0.49	0.95
C	0.23	0.06,0.48	0.01	0.36	0.13,0.61	<0.01	0.01	−0.4,0.47	0.96
D	0.23	0.06,0.46	0.01	0.35	0.11,0.61	<0.01	−0.03	−0.5,0.41	0.83

Note: Data presented as standardized β, 95% CI and associated p-values. HOMA-IR and QUICKI are indices for insulin resitance and insulin sensitivity respectively. Model A is univariate, Model B adjusted for BMI, Model C adjusted for +age, and Model D adjusted for fasting glucose. Significant at p < 0.05.

**Figure 2:**
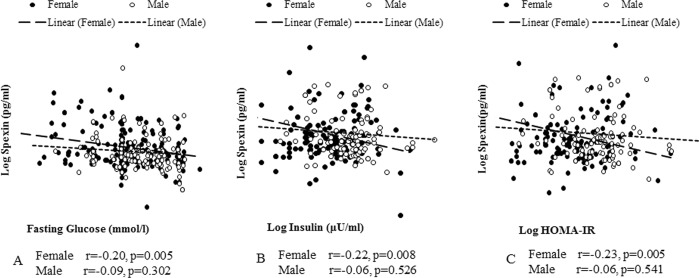
Association of spexin with glycemic indices. Fasting glucose (**A**), insulin (**B**) and HOMA-IR (**C**) on X-axis and spexin on Y-axis. Significant inverse correlations between SPX and glycemic indices were seen only in females. “r” depicts pearson correlation coefficient and “p” is the associated p-value. P-value < 0.05 is considered significant.

Figure 2 shows the associations between SPX and the glycemic indices: fasting glucose (2A), insulin (2B) and HOMA-IR (2 C) on X-axis. Significant inverse correlations between SPX and glycemic indices were seen only in females.

## Discussion

The present study investigated the circulating levels of SPX before and after a 6-month lifestyle modification program on adults with prediabetes and suggests that SPX increased significantly over time but only in women with a significant improvement in their fasting glucose. To our knowledge, the present study is the first to evaluate changes in circulating levels of SPX after a 6-month lifestyle intensive program on people with prediabetes and certainly the first in an Arab ethnic population where the prevalence of prediabetes is high^14^.

The significant decrease in fasting glucose levels in the improved group was associated with a significant increase in circulating levels of SPX. This is in accordance with previous findings that serum SPX levels are low in T2DM and correlate inversely with glucose levels^15,16^. As SPX is a relatively new biomarker, its role in humans and knowledge on the regulation of its expression is still emerging. Recent reports suggest its presence in pancreatic β cells and found negative feedback between the administration of glucose and release of SPX from pancreatic islets^17^. The mechanism driving this negative feedback is unclear as there is not much information on SPX receptors. However, these previous results and ours support the theory that SPX plays a role in glucose homeostasis and may serve as a biomarker for impaired glucose regulation seen in diabetes. The authors acknowledge that there are studies which do not support the role of SPX in glucose homeostasis^18^. This discrepancy between the results of our study and the previous one may be due to differences in study population and metabolic profile (adults with prediabetes versus normoglycemic adolescents^18^).

The significant inverse association of SPX with glucose remained even after adjustments for BMI and age. A significant inverse association of SPX was also seen with other glycemic indices like insulin and HOMA-IR, only in women, independent of fasting glucose. The significant decrease in HOMA-IR with an increase in SPX levels in this study confirms our previous work where SPX was found to be significantly lower in metabolic syndrome (MetS) group compared to the non-MetS group, particularly in women^19^, knowing that IR is a central component of MetS^20^. Our previous study on pregnant women also confirms this association only among subjects without gestational diabetes mellitus (GDM) indicating a reversal of SPX role in the presence of considerable damage to insulin-regulating mechanisms of the pancreas^21^. In both studies, the protective role of SPX as an insulin sensitizer appears only true among subjects without established diabetes. Some other studies conducted on rodents confirmed that SPX administration effectively attenuates IR^8^. The mechanism behind this attenuation of IR by SPX is not yet fully understood. However, recent studies discovered that SPX and Galanin (GAL) are in the same family of peptides and SPX binds to and activates GAL receptor 2 (GALR2) and GAL receptor 3 (GALR3)^22^, and the finding that GAL antagonist M35 significantly increased IR due to its inhibition of movement of glucose transporter 4 (GLUT4)^23,24^ provides indirect evidence for involvement of SPX in improving insulin sensitivity.

In the present study, fasting glucose levels reduced significantly in both males and females of the improved group post intervention, yet SPX levels significantly increased only in females. The sexual dimorphism observed in SPX levels post intervention may be due to the known gender differences in insulin sensitivity and glucose metabolism owing to the regulatory effects by different sex steroids^25^. Striking sexual differences in glucose metabolism have been reported in studies which conclude that obese men have higher hepatic IR than obese women^26^ which explains, in part, the higher prevalence of diabetes in men. Besides, recent studies advocate a role of SPX in fat tissue metabolism and lipid homeostasis^27^. Although lipid indices showed no correlation with circulating levels of SPX in the present study, the fact that there are sex-specific differences in lipid metabolism^28^ explains indirectly the sexual disparity observed and must be further evaluated in future studies. Several other mechanisms including the estrogen effect on IR^29^ and differences in glucoregulatory hormones such as growth hormone^30^ may explain this disparity. The sex-specific improvements in IR with an increase in SPX levels observed in this study may have clinical relevance and should be further explored.

Among the participants recruited in this study, 18.1% (n = 29) were on metformin for 6-months, aside from the lifestyle modification intervention. Metformin is known to alleviate IR mainly by improving insulin-mediated suppression of hepatic glucose production^31^. Re-analysis of data without participants on metformin (Supplementary Table 1) showed that the sex-specific increase and decrease in circulating levels of SPX and HOMA-IR, respectively, is independent of metformin supplementation.

This 6-month lifestyle intervention program focused mainly on the reduction of fasting glucose, weight loss and increase in physical activity. It resulted in a significant increase in SPX levels and a subsequent significant decrease in body weight and BMI in the improved group. These results are in line with the reports that suggest SPX is involved in weight regulation^6,9^. In fact, synthetic SPX treatment in dietary-induced obese (DIO) mice models has been consistently shown to reduce body weight possibly by its inhibition of fatty acid uptake into hepatocytes^8,32^. This has not been tried in humans yet. However, such studies, in addition to the findings observed in our data, suggest a possibility of a potential application of SPX treatment for reducing the glucose load (e.g. in impaired glucose regulation and diabetes) and obesity treatment (e.g. weight control, treatment of fatty liver, etc.) and may be useful in treating diseases such as metabolic syndrome. However, the results from many more animal and *in-vitro* studies are warranted to establish the role of this hormone in glucose and body-weight homeostasis.

Strengths of this study include its prospective design; measurement of SPX at different time intervals in the same laboratory; and the ethnic homogeneity of the study participants. The authors do acknowledge some limitations. The relatively higher number of female participants (n = 96) than males (n = 64) and the other one being its relatively short intervention period. The authors acknowledge the possibility that a non-significant increase in SPX levels in males in the improved group may be due to the small sample size. Furthermore, whether the observed elevations in SPX levels are true for DM patients with improved glycemic control cannot be ascertained. The stringent criteria for inclusion in this study limit its applicability only on people with prediabetes. Lastly, objective information on changes in dietary habits, physical activity, and body fat/muscle distribution of the study participants were not available in this study and should be evaluated in further studies. Nevertheless, this is the first study to demonstrate changes in circulating levels of SPX in people who responded favorably to a 6-month lifestyle intervention program, particularly in females.

## Conclusion

In conclusion, increased circulating SPX levels among adults with prediabetes are observed following favorable glycemic changes post-lifestyle modification program, particularly in females. Further investigations are needed using larger sample sizes to shed light whether unknown mechanisms are at play supporting the sex-dimorphic changes. The study also suggested that SPX levels correlate inversely with fasting glucose and HOMA-IR independent of age and BMI among adults with prediabetes. Our data suggests a possible role of SPX as a marker of glycemia and has potential use as anti-glycemic drug.

## Methods

### Subjects

A total of 160 subjects were randomly selected from an already existing cohort (prediabetes study) of 294 adult Saudi male and female subjects (age range 25–60) with impaired glucose regulation attending a lifestyle intervention program published previously^33^. This lifestyle intervention program was conducted by the Chair for Biomarkers of Chronic Diseases (CBCD), King Saud University (KSU) at two tertiary hospitals located in Riyadh, Saudi Arabia. The program was conducted from April 2013 till March 2017. The inclusion criteria for this program was a fasting glucose level of 5.6–6.9 mmol/l (impaired glucose regulation or prediabetes). Pregnant women and subjects with known DM; renal, hepatic, pulmonary or neurologic complications were excluded.

### Intervention

The lifestyle modification program (prediabetes study) has been previously published^33^. Briefly, eligible subjects with impaired glucose regulation had an orientation session conducted by a physician and dietician and were educated about the risk of developing T2DM. Information on their usual dietary habits and physical activity were noted and were advised some modifications in their lifestyle like increased physical activity, weight reduction of at least 5% or more, reduction of total fat intake to <30% of the energy consumed and increased fiber intake. Dietary charts, booklets, etc., prepared and translated from successful lifestyle intervention programs done elsewhere^34,35^ were distributed in order to achieve the objectives. Pedometers (081564483, Patterson Medical) were distributed to participants and advised to take at least 5000 steps per day. The intervention was monitored regularly via phone calls to the participants; however, a “self-monitoring” approach^36,37^ was largely followed.

### Anthropometry and clinical assessment

Information on anthropometrics (at baseline and 6-months) was extracted from the pre-diabetes database. Briefly, at baseline and after 6-months of intervention, subjects were advised to visit their recruiting center after an overnight fast for anthropometry and blood withdrawal by trained nurses. Anthropometry included waist and hip circumference (cm) using a standardized measuring tape. Weight (kg) and height (cm) were measured using a standardized digital scale and BMI was calculated as weight (kg)/height (m^2^). Blood pressure (mmHg) was measured twice using standard methods and the average reading was recorded. Fasting blood samples, taken at each time point, were processed immediately using standard procedures and transported to CBCD, KSU where they were aliquoted and stored at −80 °C for further analysis.

### Biochemical Measurements

Biochemical measurements for baseline and 6-months were also retrieved from the database. Fasting glucose, lipid profile, calcium and phosphorus in the samples for both time points were measured using a routine biochemistry analyzer, Konelab 20 (Thermo-Fischer Scientific, Helsinki, Finland) (catalogue# 981379 for glucose; #981812 for total cholesterol; #981823 for HDL-cholesterol; #981301 for triglycerides; #981772 for calcium and #981890 for phosphorus). The imprecision, calculated as the total CV, was between 3–5% in all tests. LDL-cholesterol was calculated using the Friedwald equation^38^. DCA vantage analyzer (Siemens, Munich, Germany) with the imprecision of <3.5% was used to measure glycated haemoglobin (HbA1c). Fasting insulin was quantified by Luminex Multiplex (Luminexcorp, Texas, USA) using fluorescent microbead technology.

For this study specifically, spexin was quantified using commercially available assay kits, supplied by Phoenix Pharmaceuticals (Burlingame, California) (catalog# EK-023-81). The imprecision for this procedure is <10% and has a 0% cross-reactivity with related human peptides like preprospexin, ghrelin, etc.

### Study groups

For the current study, 160 participants, mentioned earlier were subdivided into two groups (1:1) based on the improvement in levels of circulating fasting glucose from baseline to end of study. 80 subjects with the highest improvement in terms of reduction in fasting glucose was placed in one of the groups (improved group) and the rest of the subjects (80) was placed in the non-improved group. This division into groups was done to investigate the hypothesized change in circulating levels of SPX vis-à-vis improvement in fasting glucose. Changes in other glycemic indices like insulin, HbA1c and Homeostatic model assessment for insulin resistance (HOMA-IR) {calculated as [fasting insulin (mU/l) × fasting glucose (mmol/l)/22.5)^39^ was also seen. Besides two other indices of insulin sensitivity namely Quantitative insulin sensitivity check index (QUICKI)^40^ and McAuley insulin sensitivity index (McAuley ISI)^41^ was calculated as below:

$$\begin{array}{c}{\rm{QUICKI}}=1/\{\,\mathrm{log}({\rm{fasting}}\,{\rm{insulin}})+\,\mathrm{log}({\rm{fasting}}\,{\rm{glucose}})\}\\ {\rm{McAuley}}\,{\rm{ISI}}=\exp \{2.63-0.28\ast \,\mathrm{ln}\,({\rm{fasting}}\,{\rm{insulin}})-0.31\ast \,\mathrm{ln}\,({\rm{fasting}}\,{\rm{triglycerides}})\}\end{array}$$

**Figure 3:**
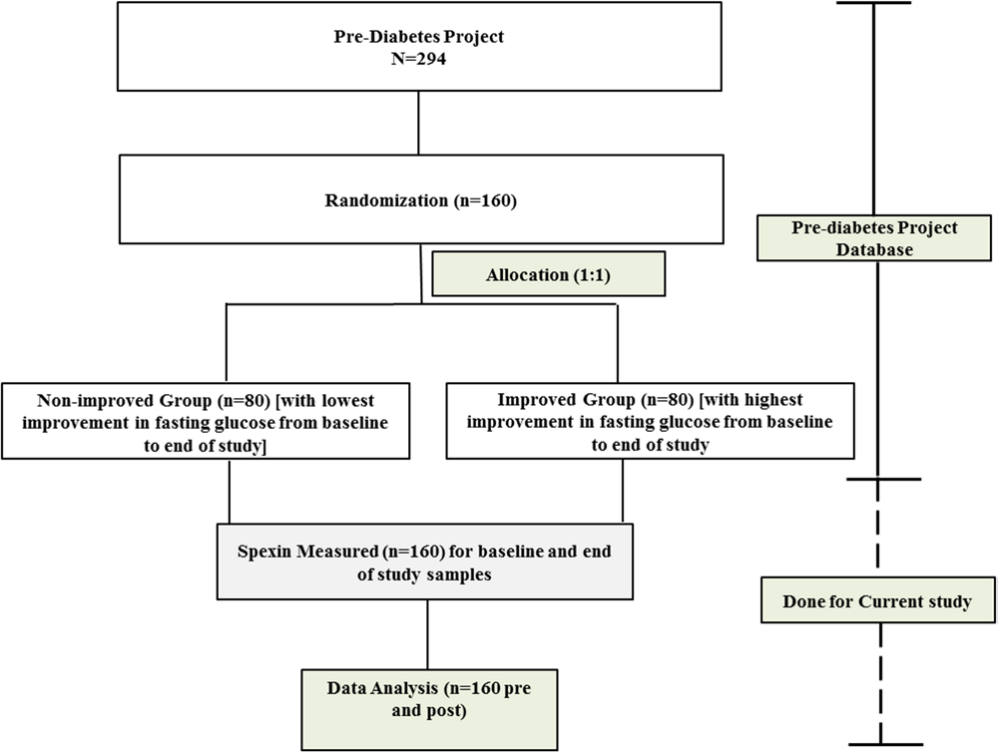
Study Flowchart.

A flowchart depicting the course of this study is provided in Fig. 3.

### Data analysis

G*Power calculator was used to determine sample size. Based on the repeated measurement analysis, the actual observed power was >0.90 for a sample size of 150. Data was analyzed using SPSS 21 (Chicago, IL, USA). Each variable was tested for normality using Shapiro-Wilk test. Continuous Gaussian variables were shown as mean ± standard deviation (SD) while continuous non-Gaussian variables were expressed as median (quartiles 1 and 3). Baseline differences between groups were calculated using independent Student T-test and Mann-Whitney U-test for normal and non-normal variables, respectively. Paired samples T-test and Wilcoxon signed-rank test were used to test differences between baseline and after 6 months, for normal and non-normal continuous variables, respectively. Non-normal variables were log-transformed before parametric analysis. Pearson correlation was used to determine associations between SPX and other variables at both time points. Two-way repeated measured ANOVA was performed to test the differences between the groups and sexes overtime. Regression analysis was done using log SPX as dependent variable and glycemic indices (fasting glucose, insulin, HbA1c, and HOMA-IR) as independent variables. Significance was set at p < 0.05. Microsoft Excel 2010 was used to plot the figures.

### Details of ethics approval

All the procedures followed in this study were in accordance with the ethical standards of the responsible committee on human experimentation (institutional and national) and with the Helsinki Declaration of 1975, as revised in 2008. The study was approved by the Ethics Committee of College of Science, King Saud University.

### Statement of informed consent

Written informed consent was obtained from each participant in this 6 month interventional study.

## Supplementary information

Supplementary Figure 1

Supplementary table 1

## Data Availability

The dataset analysed for getting the results observed in this study is available from the corresponding author on reasonable request.

## References

[1] Porzionato A (2012). Spexin is expressed in the carotid body and is upregulated by postnatal hyperoxia exposure. Adv. Exp. Med. Biol..

[2] Wong MK (2013). Goldfish spexin: solution structure and novel function as a satiety factor in feeding control. Am. J. Physiol. Endocrinol. Metab..

[3] Mirabeau O (2007). Identification of novel peptide hormones in the human proteome by hidden Markov model screening. Genome Res..

[4] Sonmez K (2009). Evolutionary sequence modeling for discovery of peptide hormones. PLoS Comput. Biol..

[5] Porzionato A (2010). Spexin expression in normal rat tissues. J. Histochem. Cytochem..

[6] Walewski JL (2014). Spexin is a novel human peptide that reduces adipocyte uptake of long chain fatty acids and causes weight loss in rodents with diet‐induced obesity. Obesity.

[7] Wu H (2016). Ya-fish (Schizothorax prenanti) spexin: identification, tissue distribution and mRNA expression responses to periprandial and fasting. Fish Physiol. Biochem..

[8] Jasmine FG, Walewski J, Anglade D, Berk P (2016). Regulation of hepatocellular fatty acid uptake in mouse models of fatty liver disease with and without functional leptin signaling: roles of NfKB and SREBP-1C and the effects of spexin. Semin. Liver Dis..

[9] Kumar S (2016). Decreased circulating levels of spexin in obese children. J. Clin. Endocrinol. Metab..

[10] Kołodziejski P (2018). Serum levels of spexin and kisspeptin negatively correlate with obesity and insulin resistance in women. Physiol. Res..

[11] Group DPPR (2002). Reduction in the incidence of type 2 diabetes with lifestyle intervention or metformin. N. Engl. J. Med..

[12] Pan X-R (1997). Effects of diet and exercise in preventing NIDDM in people with impaired glucose tolerance: the Da Qing IGT and Diabetes Study. Diabetes Care.

[13] Ramachandran A (2006). The Indian Diabetes Prevention Programme shows that lifestyle modification and metformin prevent type 2 diabetes in Asian Indian subjects with impaired glucose tolerance (IDPP-1). Diabetologia.

[14] Bahijri SM, Jambi HA, Al Raddadi RM, Ferns G, Tuomilehto J (2016). The prevalence of diabetes and prediabetes in the adult population of Jeddah, Saudi Arabia-a community-based survey. PLoS One.

[15] Gu L (2015). Spexin peptide is expressed in human endocrine and epithelial tissues and reduced after glucose load in type 2 diabetes. Peptides.

[16] Lin C-y (2018). Circulating spexin levels negatively correlate with age, BMI, fasting glucose, and triglycerides in healthy adult women. J. Endocr. Soc..

[17] Sassek, M., Kolodziejski, P. A., Szczepankiewicz, D. & Pruszynska-Oszmalek, E. Spexin in the physiology of pancreatic islets—mutual interactions with insulin. *Endocrine*, 1–7 (2018). 10.1007/s12020-018-1766-2 30267353

[18] Hodges SK, Teague AM, Dasari PS, Short KR (2018). Effect of obesity and type 2 diabetes, and glucose ingestion on circulating spexin concentration in adolescents. Pediatr. Diabetes..

[19] Al-Daghri, N. M. *et al*. Spexin Levels Are Associated with Metabolic Syndrome Components. *Dis*. *Markers* **2018** (2018). 10.1155/2018/1679690 PMC6142736 30254709

[20] Hanson RL, Imperatore G, Bennett PH, Knowler WC (2002). Components of the “metabolic syndrome” and incidence of type 2 diabetes. Diabetes.

[21] Al-Daghri NM (2018). Circulating spexin levels are influenced by the presence or absence of gestational diabetes. Cytokine.

[22] Reyes-Alcaraz A (2016). Development of spexin-based human galanin receptor type II-specific agonists with increased stability in serum and anxiolytic effect in mice. Sci. Rep..

[23] Guo L (2011). Galanin antagonist increases insulin resistance by reducing glucose transporter 4 effect in adipocytes of rats. Gen. Comp. Endocrinol..

[24] He B (2011). Beneficial effect of galanin on insulin sensitivity in muscle of type 2 diabetic rats. Physiol. Behav..

[25] Macotela, Y., Boucher, J., Tran, T. T., Gesta, S. & Kahn, C. Gender and Depot Differences in Adipocyte Insulin Sensitivity and the Sexual Dimorphism of Insulin Resistance. *Diabetes* **56** (2007). 10.2337/db08-1054 PMC2661589 19136652

[26] Ter Horst KW (2015). Sexual dimorphism in hepatic, adipose tissue, and peripheral tissue insulin sensitivity in obese humans. Front Endocrinol.

[27] Kolodziejski PA (2018). Spexin: A novel regulator of adipogenesis and fat tissue metabolism. Biochim Biophys Acta Mol Cell Biol Lipids.

[28] Varlamov O, Bethea CL, Roberts CT (2015). Sex-specific differences in lipid and glucose metabolism. Fron Endocrinol.

[29] Zhu L (2013). Estrogen treatment after ovariectomy protects against fatty liver and may improve pathway-selective insulin resistance. Diabetes.

[30] Jeffcoate W (2002). Growth Hormone Therapy and its Relationship to Insulin Resistance, Glucose Intolerance and Diabetes Mellitus. Drug safety.

[31] Giannarelli R, Aragona M, Coppelli A, Del Prato S (2003). Reducing insulin resistance with metformin: the evidence today. Diabetes. Metab..

[32] Lin C-y (2018). Spexin Acts as Novel Regulator for Bile Acid Synthesis. Front. Physiol..

[33] Alfawaz HA (2018). Effects of Different Dietary and Lifestyle Modification Therapies on Metabolic Syndrome in Prediabetic Arab Patients: A 12-Month Longitudinal Study. Nutrients.

[34] Tuomilehto J (2001). Prevention of type 2 diabetes mellitus by changes in lifestyle among subjects with impaired glucose tolerance. N. Engl. J. Med..

[35] Kosaka K, Noda M, Kuzuya T (2005). Prevention of type 2 diabetes by lifestyle intervention: a Japanese trial in IGT males. Diabetes Res. Clin. Prac..

[36] Al-Daghri NM (2014). A 6-month “self-monitoring” lifestyle modification with increased sunlight exposure modestly improves vitamin D status, lipid profile and glycemic status in overweight and obese Saudi adults with varying glycemic levels. Lipids Health Disease.

[37] Al-Daghri NM (2016). Age-specific improvements in impaired fasting glucose and vitamin D status using a lifestyle intervention programme in overweight and obese Saudi subjects. Int. J. Clin. Exp. Med..

[38] Friedewald WT, Levy RI, Fredrickson DS (1972). Estimation of the concentration of low-density lipoprotein cholesterol in plasma, without use of the preparative ultracentrifuge. Clin. Chem..

[39] Bonora E (2002). HOMA-estimated insulin resistance is an independent predictor of cardiovascular disease in type 2 diabetic subjects: prospective data from the Verona Diabetes Complications Study. Diabetes Care.

[40] Katz A (2000). Quantitative insulin sensitivity check index: a simple, accurate method for assessing insulin sensitivity in humans. J Clin Endocrinol Metab.

[41] McAuley KA (2001). Diagnosing insulin resistance in the general population. Diabetes care.

