# Supplementary material for: Favorable Changes in Fasting Glucose in a 6-month Self-Monitored Lifestyle Modification Programme Inversely Affects Spexin Levels in Females with Prediabetes
Source: Sci Rep. 2019 Jul 1;9:9454. doi: 10.1038/s41598-019-46006-0 (PMC6602932; doi:10.1038/s41598-019-46006-0)
Supplement: Supplementary file 1 — Supplementary Figure 1 [file 41598_2019_46006_MOESM1_ESM.docx]

Favorable Changes in Fasting Glucose in a 6-month Self-Monitored Lifestyle Modification Programme Inversely Affects Spexin Levels in Females with Prediabetes

Nasser M. Al-Daghri, Kaiser Wani, Sobhy M. Yakout, Hazim Al-Hazmi**,** Osama E. Amer, Syed Danish Hussain, Shaun Sabico, Mohammed Ghouse Ahmed Ansari, Sara Al-Musharaf, Amal M. Alenad, Majed S. Alokail, Mario Clerici

**Supplementary Figure 1:** Circulating levels of SPX at baseline and overtime in tertiles based on improvement in FG.


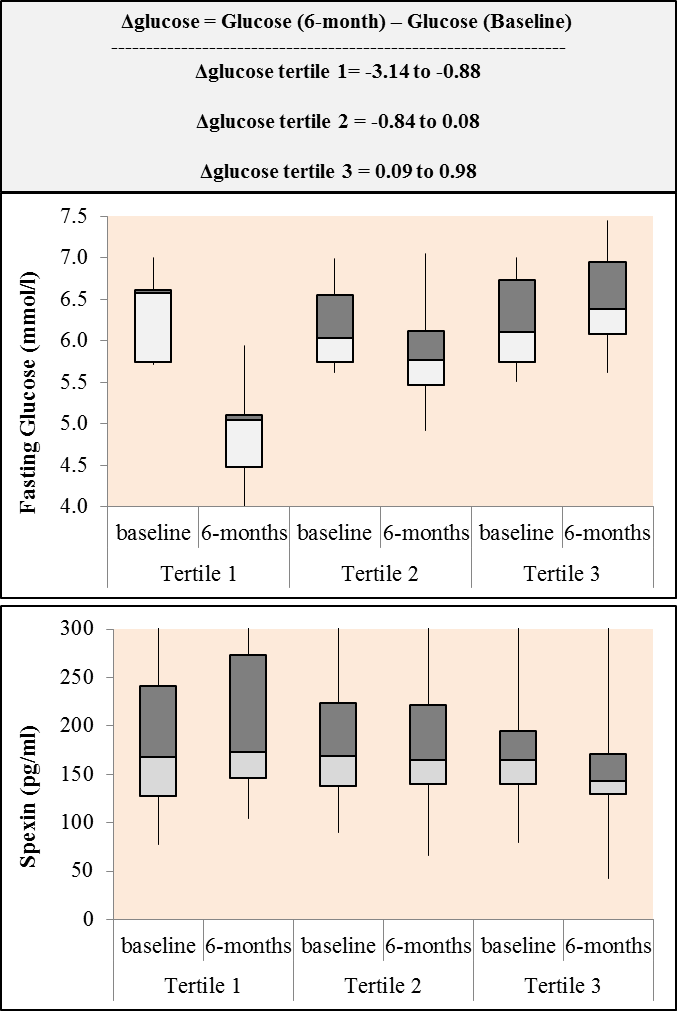


Note: Data from all subjects was sorted according to the improvement in FG (FG at end of study – FG at baseline) and divided into three parts as T1, T2 and T3 with T1 and T3 being the ones where most and least improvement was seen respectively.
