# Supplementary material for: Favorable Changes in Fasting Glucose in a 6-month Self-Monitored Lifestyle Modification Programme Inversely Affects Spexin Levels in Females with Prediabetes
Source: Sci Rep. 2019 Jul 1;9:9454. doi: 10.1038/s41598-019-46006-0 (PMC6602932; doi:10.1038/s41598-019-46006-0)
Supplement: Supplementary file 2 — Supplementary table 1 [file 41598_2019_46006_MOESM2_ESM.docx]

Favorable Changes in Fasting Glucose in a 6-month Self-Monitored Lifestyle Modification Programme Inversely Affects Spexin Levels in Females with Prediabetes

Nasser M. Al-Daghri, Kaiser Wani, Sobhy M. Yakout, Hazim Al-Hazmi**,** Osama E. Amer, Syed Danish Hussain, Shaun Sabico, Mohammed Ghouse Ahmed Ansari, Sara Al-Musharaf, Amal M. Alenad, Majed S. Alokail, Mario Clerici

**Supplementary table 1:** Sex specific changes in clinical characteristics in the two study groups (After removing the ones who were on metformin supplementation for six months).

| Biochemical Parameters | Non-improved Group (63) | | | | Improved Group (68) | | | |
| --- | --- | --- | --- | --- | --- | --- | --- | --- |
|  | Female (28) | | Male (35) | | Female (43) | | Male (25) | |
|  | Baseline | 6-months | Baseline | 6-months | Baseline | 6-months | Baseline | 6-months |
| Anthropometrics | | | | | | | | |
| Weight (Kg) | 78.24 ± 7.6 | 80.12 ± 9.1 | 80.6 ± 13.5 | 82.85 ± 15 | 75.98 ± 15 | 74.77 ± 14.5 | 87.02 ± 11 | 85.09 ± 11** |
| BMI (Kg/m^2^) | 32.31 ± 4.1 | 33.13 ± 5 | 29.73 ± 4.9 | 30.66 ± 6.1 | 31.47 ± 5.9 | 30.99 ± 5.8 | 30.53 ± 4.6 | 29.88 ± 4.8** |
| Waist (cm) | 97.08 ± 11.5 | 96.19 ± 11.6 | 106.47 ± 10.7 | 107.94 ± 10.2 | 93.19 ± 12.6 | 92.21 ± 12.8* | 107.44 ± 8.5 | 108.31 ± 8.6 |
| Hips (cm) | 111.54 ± 6 | 110.28 ± 6.6 | 107.83 ± 12.6 | 106.55 ± 10.5 | 107.58 ± 12.8 | 107.7 ± 12.1 | 105.57 ± 7.4 | 104.43 ± 9.7 |
| Systolic BP (mmHG) | 124.32 ± 15.4 | 123.73 ± 17.8 | 123.85 ± 9.6 | 125.95 ± 12.7 | 120.15 ± 14.8 | 115.2 ± 19.3 | 126.96 ± 17 | 124.82 ± 12 |
| Disystolic BP (mmHG) | 78.61 ± 12.3 | 76.5 ± 12.4 | 75.48 ± 7.2 | 79.23 ± 9.5** | 77.06 ± 11.8 | 76.28 ± 13.6 | 81.38 ± 8.6 | 77.18 ± 7.7 |
| Glycemic Indexes | | | | | | | | |
| Fasting Glucose (mmol/l) | 6.4 ± 0.5 | 6.4 ± 0.5 | 6.25 ± 0.5 | 6.28 ± 0.5 | 5.98 ± 0.4 | 4.85 ± 0.6** | 6.45 ± 0.3 | 5.06 ± 0.2** |
| Insulin (μU/ml) | 11.26 (5.8,24.5 ) | 9.2 (6,14.5 ) | 16.35 (11.1,28.9 ) | 19.43 ± 11.3 | 14.42 (5.2,28 ) | 9.73 (3.1,18.4 )* | 27.19 (14.6,39.2 ) | 27.13 (19.8,43.4 ) |
| HbA1c (%) | 5.86 ± 0.4 | 6.27 ± 0.8 | 5.95 ± 1.3 | 6.26 ± 1 | 5.57 ± 0.2 | 4.8 ± 1.2** | 6.22 ± 0.6 | 5.35 ± 2* |
| HOMA_IR | 3.23 (1.7,7.4 ) | 2.54 (1.8,3.8 ) | 4.32 (3,7.6 ) | 4.4 (3.1,7.8 ) | 3.68 (1.3,7.1 ) | 2.12 (0.7,3.6 )** | 7.55 (3.8,11.5 ) | 6.15 (4.5,9.3 ) |
| SPX (Pg/ml) | 169.5 (138.5,201.5 ) | 153 (137.5,174 ) | 163 (134,197 ) | 140 (122,180 ) | 169 (127,252 ) | 187 (151,369.1 )** | 165 (144,205 ) | 171 (145,199 ) |

Note: Data presented as Mean ± SD for continuous normal variables and medians (25^th^ percentile, 75^th^ percentile) for continuous non-normal variables. Paired samples t-test and Wilcoxon signed-rank test is used to test the differences in central tendency for continous normal and non-normal variables respectively. FBG and SPX referes to fasting blood glucose and Spexin respectively. P<0.05 is taken as significant. * depicts p-value <0.05 and ** depicts p-value <0.01.
